# Medical and Surgical Memoirs

**Published:** 1887-04

**Authors:** 


MEDICAL AND SURGICAL MEMOIRS. Containing investigations on geographical distribution, causes, nature, relations and treatment of various diseases. 1885 and 1886. By Joseph Jones, M. D., Professor of Chemistry, Clinical Medicine, etc., in the University of Louisiana.

To do anything like justice to the claims of this most valuable contribution to medical science, or appropriately commend it to professional favor, would require more space than the limits of your journal afford. Nor does the writer flatter himself that any expressions of approval or commendation of which he is capable will give other than a faint idea of its intrinsic merits, which alone can be estimated by those who shall do themselves and its distinguished author justice by consulting its rich contents. Any selection or mention we may make from its many attractive features will be but as a drop to the ocean's fullness and serve but as a taste to the inviting feast in store for its readers.

As a guarantee as to the nature and quality of this contribution to medical science, we quote the author's views on this all-important head. Says he: "We have reason and hypothesis in abundance; medical science now demands facts. These," he says, "cannot be obtained by legislative enactments, nor by windy discussions in conventions and committees, where the superficial, brazen-faced egotists most generally succeed in establishing so-called conclusions."

The author has not only erected the true standard, but has strictly complied with its just demands, which, in a large majority of the so-called contributions to medical science of the present day, is more "honored in the breach than in the observance." The author has enjoyed every facility for the acquisition of information on the subjects of which he treats. In addition to which he possesses by nature and education a mind pre-eminently qualified for the task, and accustomed to close and analytical investigations. With abundant material supplied by his large and extended practice, and supplied with every facility and instrument known to science, and with an unequalled skill in their use, we are prepared to accept his conclusions as amounting to almost mathematical certainty. We must, however, curb our desire to further compliment the distinguished author, or to commend his worth, as anything we may say will fall far short of our true estimate of the well-earned fame of the former, or of the just claims of the atter to professional favor. We refer briefly to a few of its leading excellencies and special attractions, among which we venture to mention the tables giving the composition of human blood and of other warm-blooded animals, and contrasting and comparing the same, giving the peculiarities of each, and how to differentiate them; also comparing air-breathing with amphibious animals in the composition of their blood and its peculiarities in each; also showing the rate of the heart's action, respiratory movements, temperature, and so on, from which much that is new and valuable may be gathered. How to detect and determine blood-stains will be found of inestimable advantage in a medico-legal sense and in expert testimony in criminal prosecutions. The changes wrought in the blood by disease are most interestingly stated, as well as the changes peculiar to special disease, such as malarial fevers, will challenge the attention of the thoughtful inquirer, as must also the means and mode of detecting sugar and other foreign elements in the blood. The author's contributions on these heads will no doubt rank as authority in medico-legal investigations, and as a work of reference should find a place in every library.

The articles on malaria, hæmaturic, typhoid and yellow fevers will richly repay their careful perusal, and will be found replete with much that is new and highly instructive. On the subject of quarentine, the author's views are abreast of the most advanced workers and writers on hygiene, and especially is this true on the question of preventing the introduction of yellow fever. Dr. Jones' labors in this branch alone entitle him to a front place in the ranks of the most noted benefactors of the day. Under his judicious teachings and acts as President of the State Board of Health of Louisiana, the death-rate has been reduced in New Orleans to only a fraction of what it was before his wise counsels prevailed. His contributions and labors on the subject of yellow fever have received the grateful recognition of his State and city of his home, and give to his utterances on this most important department more than ordinary weight and authority.

On the digestion of albumen and flesh, we fine much that is new and instructive, as we do in his remarks on the anatomy and physiology of the pancreas.

We might and could extend this notice indefinitely, and still leave the most interesting features of this book unnoticed. We cannot, however, close this imperfect notice without calling attention to the articles on elephantiasis and leprosy. These are two diseases which it seldom falls to the lot of the physician in this country to treat, and yet the doctor has been favored with cases of each, with which he has enriched this book, and furnished accurate delineations and drawings. The descriptions and drawings of the various animalculæ supposed to propagate disease are most graphically portrayed and described, as well as the rapidity with which they multiply and propagate. Without saying more, we close by expressing the hope that this new candidate for public favor may meet with a patronage commensurate with its merits, and that it may find a place in every medical library as a work of reference, as it cannot be surpassed in this respect by any work on the subjects of which it treats. T. J. W.

